# Mural Unicystic Ameloblastoma of the Mandible: A Case Report

**DOI:** 10.3390/reports7040093

**Published:** 2024-11-07

**Authors:** Mina Al Azawi, Nikolaos Shinas, Vasileios Zisis, Dhurata Shosho, Athanasios Poulopoulos, Deeba Kashtwari

**Affiliations:** 1 Department of Oral and Maxillofacial Radiology, Henry M. Goldman School of Dental Medicine, Boston University, Boston, MA 02215, USAshinas@bu.edu (N.S.); dhurata@bu.edu (D.S.);; 2 Department of Oral Medicine/Pathology, Dental School, Aristotle University of Thessaloniki, 541 24 Thessaloniki, Greece

**Keywords:** CBCT, jaw tumors, mural, unicystic ameloblastoma

## Abstract

**Background and Clinical Significance**: Among the odontogenic tumors, ameloblastoma is one of the most notorious, although it remains relatively rare, accounting for approximately one percent of all oral tumors. This neoplasm, derived from odontogenic epithelium, may arise from the developing enamel organ, epithelial cell rests of dental lamina, epithelial lining of odontogenic cysts, and basal cells of oral epithelium. This is a case presentation of a mural unicystic ameloblastoma, the most aggressive subtype and the one with the highest chance of recurrence. **Case Presentation**: A patient was referred by his dentist for root canal treatment at the Emergency Dental Clinic of Boston University. The patient complained of mandibular numbness. A panoramic radiograph was acquired, revealing a radiolucent lesion in the right mandible. Clinical examination detected a soft swelling perforating the buccal cortex in the area of #27–#30. A Cone-Beam CT (CBCT) was acquired in the Oral and Maxillofacial Radiology Clinic revealing a well-defined, partially corticated entity in the periapical area of teeth #27 through #30, with evidence of scalloping borders. The internal structure was unilocular and uniformly low-density. The entity caused interruption of the lamina dura of the associated teeth and inferior displacement of the inferior alveolar canal. Differential diagnoses included unicystic ameloblastoma (UA) and central giant cell granuloma as a second less likely diagnosis. An incisional biopsy was performed for further evaluation. Biopsy confirmed UA with mural involvement. **Conclusions**: UAs typically exhibit less aggressive behavior. However, cases like this one, where mural involvement is noted and no associated impaction is detected, underline the possibility of variable radiographic presentation and the significance of a multidisciplinary approach in correct diagnosis and treatment. Histological subtyping is crucial for guiding treatment.

## 1. Introduction and Clinical Significance

Over the past five decades, odontogenic tumors have been studied extensively. The World Health Organization (WHO) revises the classification of such lesions every few years [1,2], with the most recent 2022 edition being fifth in order.

Among the odontogenic tumors, ameloblastoma is one of the most notorious, although it remains relatively rare, accounting for approximately one percent of all oral tumors. This neoplasm, derived from odontogenic epithelium, may arise from the developing enamel organ, epithelial cell rests of dental lamina, epithelial lining of odontogenic cysts, and basal cells of oral epithelium. It is a benign tumor but exhibits aggressive characteristics, including persistent growth and local invasiveness [3].

These tumors pose significant challenges in clinical management due to their propensity for recurrence. Factors influencing recurrence include tumor subtype, treatment modality, and tumor behavior [2].

The prevalence of ameloblastoma is notably higher in Asian and African populations, whereas it is less common in North American and European countries. Even though it has been established worldwide, demographic profiles and histopathological data in different populations remain inadequately detailed [3].

Ameloblastomas encompass a range of histopathological subtypes, presenting a challenging spectrum of odontogenic tumors [3,4]; however, classifications provide clinicians with valuable insights into tumor behavior and guide treatment decision-making [5,6].

There are three basic types of ameloblastomas: conventional, peripheral, and unicystic [1]. Unicystic ameloblastoma (UA) stands out as a distinct entity, accounting for a significant proportion of cases [4,7]. Histologically, it is characterized by ameloblastomatous epithelium lining part of the cyst cavity, distinguishing it from other cystic lesions. UA typically manifests as a unilocular radiolucency in the jaw, posing diagnostic challenges due to its resemblance to cystic lesions. This variant often affects younger individuals and responds well to conservative surgical approaches [7]. It exhibits diverse histological patterns with significant prognostic implications [5].

Ackermann et al. in 1988 [8] proposed three distinct types of UA based on histological features, emphasizing the importance of distinguishing between various subtypes based on histological features and their implications for treatment.

The luminal UA comprises unilocular cystic lesions lined by ameloblastomatous epithelium [4,5,8]. Inactive rests of odontogenic origin may be present but there is no evidence of infiltration of neoplastic epithelium [8]. This type typically presents a more contained growth pattern, making it amenable to conservative surgical approaches with a lower risk of recurrence [4,5,7]. However, enucleation may be sufficient only for tumors that have proliferated into the lumen, while subtypes involving the periphery of the cyst wall must be treated more radically, like solid or multicystic ameloblastoma [6].

Intraluminal UA demonstrates features of the luminal type but also includes intraluminal proliferation without infiltration into the connective tissue wall of the cyst [5,6]. It has epithelial nodules arising from the cystic lining, projecting into the cyst lumen [3,8]. These nodules comprise epithelium with a plexiform or follicular pattern resembling that seen in intraosseous ameloblastoma [5]. While still confined to the cyst lining, the presence of intraluminal proliferation in this type may require more cautious management strategies as it may present a higher risk of recurrence compared to the luminal variant, particularly if the proliferation is extensive or not completely removed during surgical intervention [6].

Mural UA is characterized by the presence of invasive islands of ameloblastomatous epithelium in the connective tissue wall of the cyst [5]. These islands may or may not be connected to the cyst lining, indicating infiltration into the surrounding tissue [3,4,8]. Mural UA poses the highest risk of recurrence and requires more aggressive treatment approaches, akin to solid or multicystic ameloblastoma, to ensure complete eradication of the tumor [5,9].

It is essential to understand UA subtypes and to distinguish between those with intraluminal proliferation and those with invasive islands of ameloblastomatous epithelium in the connective tissue wall of the cyst [9]. While luminal UA may be managed conservatively with enucleation or curettage, intraluminal and mural UAs may necessitate more radical surgical interventions to achieve complete excision and reduce the risk of recurrence [5,6].

The following is a case report of a unicystic ameloblastoma of the mandible.

## 2. Case Presentation

A waiver of authorization was requested by the authors, and granted by the Compliance Office and Institutional Review Board (IRB) of Boston University Henry M. Goldman School of Dental Medicine to release patient information, after careful consideration that anonymity has been achieved. For that reason, some information about the patient has been deducted.

**Figure 1:**
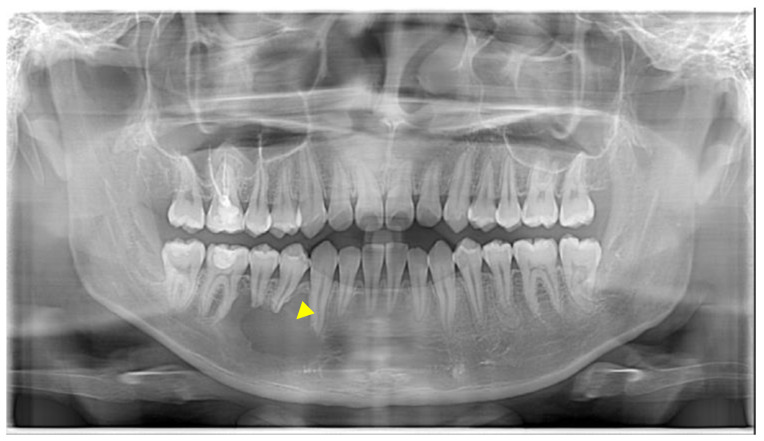
The yellow arrowhead shows a unilocular lesion detected in the area of #27–30.

A young male presented in the Urgent Care Clinic at Boston University Henry M. Goldman School of Dental Medicine with a panoramic radiograph, seeking dental care due to numbness in the lower right region of his mandible. His dentist had referred him for a root canal treatment (Figure 1).

**Figure 2:**
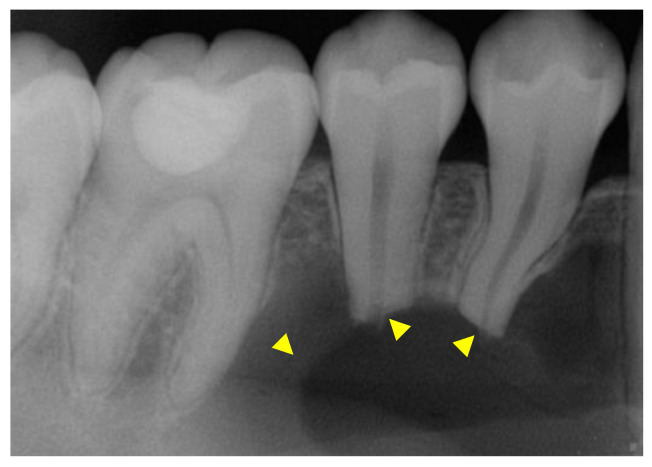
The yellow arrowheads show the unilocular intrabony lesion. There is evidence of root resorption in the apical third of the roots.

After the initial clinical examination, an intraoral periapical radiograph was acquired (Figure 2) and the patient was referred to Oral and Maxillofacial Surgery (OS) for further evaluation.

A detailed history in the Department of Oral Surgery (OS) revealed that the patient was experiencing pain upon palpation on the right side of the mandible for the past month and discomfort while chewing on the same side for two weeks. His general dentist referred him after noting a moderately sized unilocular, scalloping radiolucency associated with teeth #27, #28, #29, and #30 on the panoramic radiograph. At the OS clinic, the patient underwent a thorough extraoral examination, which showed no sign of swelling, and exhibited a full range of motion of his mandible. Intraoral examination revealed a palpable soft, squishy swelling perforating the buccal cortex in the area of #27–#30 with all teeth in the lower right quadrant evaluated as vital. The neurological assessment revealed intact cranial nerves (CN) V1 and V2, but the right CN V3 distribution in the right chin area exhibited paresthesia.

**Figure 3:**
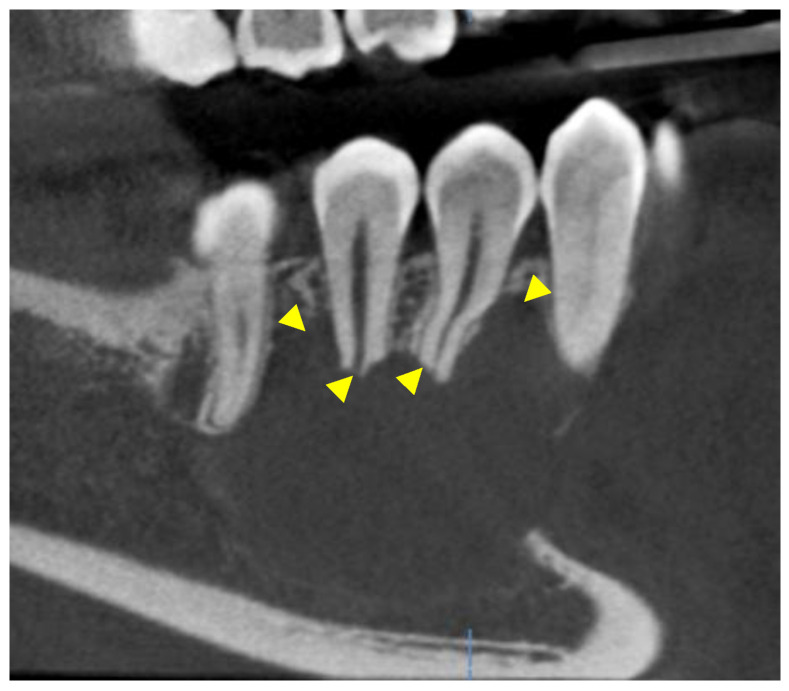
Oblique sagittal views on a CBCT study. The yellow arrowheads show evidence of root resorption and scalloping between the roots of the involved teeth.

**Figure 4:**
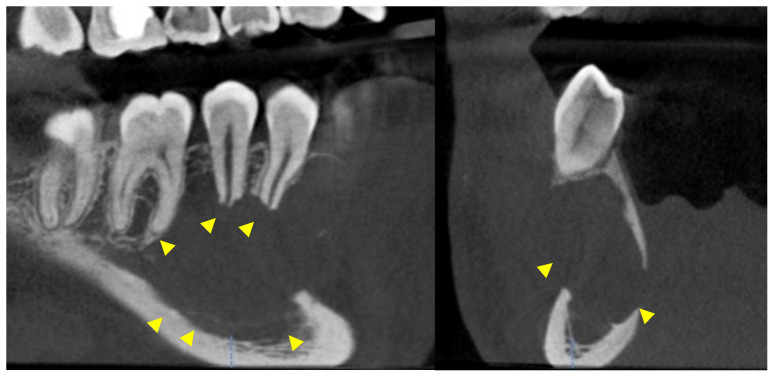
Oblique sagittal and cross-sectional views on a CBCT study. The yellow arrowheads point to the interruption of the lamina dura, the inferior displacement of the inferior alveolar canal, and the thinning/expansion/interruption of the buccal and lingual cortices.

**Figure 5:**
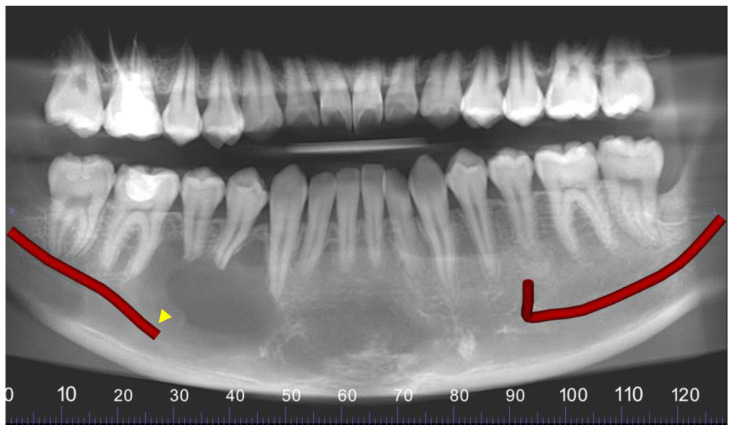
Panoramic reconstruction of the volume. The right inferior alveolar canal is interrupted and displaced inferiorly (yellow arrowhead).

Further investigation with a cone-beam computed tomography (CBCT) was scheduled and a scan was acquired using DENTSPLY Sirona© XG3D with a Field Of View (FOV) of 8 cm width and 5 cm height. The exposure settings were set at 85 kilovoltage peak (kVp), 6 milliamperes (mA), and 14.179 s (sec). The scan confirmed the presence of a moderate to large entity in the right posterior mandible. Mesiodistally, the entity extended from the distal aspect of the root of #27 to the mesial aspect of the mesial root of #30. It extended from the level of the alveolar crest and the apical half of the roots of the aforementioned teeth to a few millimeters superior to the inferior cortical border of the mandible. Bucco-lingually, the entity covered the entire width of the mandible. The entity was well defined, unilocular, and partially corticated, with evidence of scalloping between the roots of teeth #27, 28, 29, and #30. Evidence of interruption of the lamina dura and moderate root resorption was noted in the apical half of the roots of the associated teeth. Non-uniform thinning, expansion, and interruption of the buccal and lingual cortices were detected and the inferior alveolar canal was interrupted and inferiorly displaced (Figure 3, Figure 4 and Figure 5).

Radiographic findings were highly suggestive of a unicystic ameloblastoma or, less likely, central giant cell granuloma (CGCG). Typically, CGCGs are characterized by more expansion, with thin and wispy septations [10]; the internal structure, due to reactive histopathological features, shows evidence of grainy and reactive bone [11]. These findings indicated the need for further evaluation with a biopsy to confirm the diagnosis and determine appropriate management. The patient was informed about the findings, treatment plan, and postoperative care, to which he consented.

An incisional biopsy was performed to obtain a tissue sample for histopathological examination. After administering local anesthesia using three capsules of 2% Lidocaine with 1:100 k epinephrine via inferior alveolar nerve (IAN), long buccal, and mental blocks, along with local infiltration techniques, anesthesia was achieved. A #15 blade was then used to make an intrasulcular incision extending from tooth #26 to #31 without a release incision. Subsequently, a periosteal elevator was utilized to reflect a buccal full-thickness mucoperiosteal flap, revealing erosion of the buccal cortex in the region of teeth #27–29. Part of the epithelial lining was excised, and the specimen was placed in a formalin solution for further examination. Following irrigation of the area, primary closure was performed using 3-0 chromic gut sutures, ensuring hemostasis. The patient tolerated the procedure well without any complications. A regimen of Tylenol/Ibuprofen combination was recommended for pain management. The excised tissue specimen was sent to the pathology lab for analysis.

**Figure 6:**
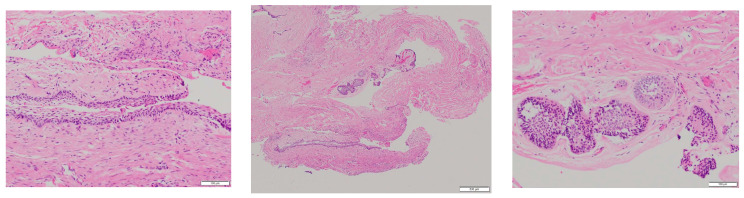
H&E (low and medium power views, (100 μm, 500 μm, 100 μm), from **left** to **right**): The cyst epithelial lining shows ameloblastic differentiation with hyperchromatic basal epithelial cells exhibiting reverse polarization. Neoplastic epithelial islands exhibiting ameloblastic differentiation are noted in the connective tissue consistent with mural involvement.

**Figure 7:**
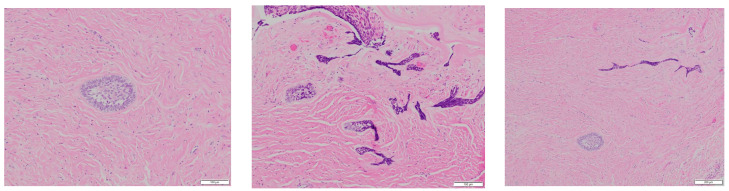
H&E (medium and high power views, (100 μm, 100 μm, 200 μm), from **left** to **right**): Scattered epithelial islands exhibiting ameloblastic differentiation are noted in the connective tissue wall of the cyst consistent with mural involvement. The peripheral cuboidal or columnar cells in the epithelial island appear hyperchromatic and exhibit reverse polarization. The central cells are loosely arranged mimicking the stellate reticulum.

The specimen showed evidence of cyst epithelial lining that underwent ameloblastic differentiation and scattered islands of neoplastic epithelial cells in the adjacent connective tissue, thus proving mural involvement (Figure 6 and Figure 7).

The subsequent pathology report confirmed the presence of a unicystic ameloblastoma with mural involvement extending to tissue edges. Management decisions were made based on these findings, with further treatment planned accordingly. The patient opted to seek a second opinion outside of the Boston University institution to confirm the diagnosis.

## 3. Discussion

First described as a distinct entity by Robinson and Martinez in 1977 [12], UA comprises 5% to 22% of all diagnosed ameloblastoma cases [13]. It commonly affects patients between the second to third decades of life, with a slight male predilection, and is predominantly found in the posterior region of the mandible, often presenting as a unilocular radiographic image. Unilocular ameloblastomas tend to occur in younger age groups, with 59.2% of cases fitting this pattern and a marked predilection for the mandible, occurring in 93.9% of cases [14]. The mean age at diagnosis for intraosseous ameloblastomas has been reported to be 39 years, but younger mean ages have been documented, potentially reflecting ethnic differences or differences in healthcare systems. Histological patterns also vary, with the plexiform pattern being most prevalent in some studies, contrasting with others where the follicular pattern predominates. This variation highlights the need for thorough histopathological evaluation to guide treatment.

Radiographically, UAs present as well-defined low-density entities and, as the term “unicystic” suggests, are predominantly unilocular and rarely multilocular [15]. On the other hand, the classic/multicystic ameloblastoma has the characteristic “soap bubble” appearance [16]. Features such as buccal and lingual cortical plate expansion and tooth and nobble structure displacement are common findings.

The comprehensive evaluation of UA is paramount for accurate diagnosis and effective management. UA, a variant of ameloblastoma, exhibits relatively benign behavior and a better response to conservative treatment. Due to its clinical and radiological similarity to cysts, accurate diagnosis of UA often requires careful histological examination, as the entire cystic lining may not uniformly exhibit characteristic features. Multiple biopsies from large cystic lesions are recommended to ensure representative sampling, paying attention to luminal, intraluminal, and mural subtypes. Meticulous histological subtyping is pivotal in guiding treatment decisions, as mural involvement correlates with a slightly higher recurrence rate. Despite the predominantly favorable prognosis of UA, with recurrence rates ranging from 10% to 25%, there remains a need for follow-up to monitor for any signs of recurrence [17]. Conservative treatment approaches in managing UA are important, particularly in pediatric patients, to mitigate the risk of facial deformity and functional impairment associated with more aggressive surgical interventions [10,11]. Further research, including large-scale prospective studies, is warranted to elucidate optimal management strategies and refine prognostic indicators for UA [17].

Histological subtyping is crucial, with mural involvement correlating with a slightly higher recurrence rate [5]. Despite the predominantly favorable prognosis of UA, recurrence rates range from 10% to 25%, particularly in cases with mural invasion [7]. While conservative treatment approaches are preferable, the choice of treatment modality significantly influences recurrence rates, with reported rates varying from 10% to 40% after conservative treatment. Resection remains the most effective method for minimizing recurrence risk, although its use is reserved for specific cases due to associated morbidity [18]. Regular follow-up is imperative, as recurrence may manifest years after initial treatment [7]. Thus, an integrative approach that considers histological subtypes, treatment modalities, and vigilant follow-up is essential for managing UA effectively.

In the current series by Figueiredo et al. 2014 [19], 11 cases of ameloblastoma were analyzed, revealing a higher incidence in females with a male-to-female ratio of 1:1.75. The youngest patient was 15 years old, while the eldest was 69 years, with a mean age of 23.25 years for males and 43.43 years for females, showing a significant difference in age of occurrence between genders. Most ameloblastomas (90.9%) occurred in the mandible, particularly in the posterior mandible. Histopathologically, the series included solid/multicystic ameloblastomas, unicystic ameloblastomas, and one desmoplastic variant. Treatment varied from conservative surgical therapy to radical surgery, with no signs of recurrence during the follow-up period.

Regarding treatment options, UA management spans from conservative to radical approaches. Conservative modalities include marsupialization, enucleation, and curettage with or without adjuvant therapies, while radical options encompass marginal or segmental resection [13]. The choice of treatment modality depends on factors such as lesion size, location, patient’s age, surgeon’s practice, and patient’s decision.

A comprehensive review of 616 reported cases of ameloblastoma over a 13-year period revealed that 5% of the cases were diagnosed as ameloblastoma, with a mean age of 31.3 years and a slight male predominance [3]. The majority of cases were found in the mandible (86.7%), particularly in the posterior region (63.3%). These findings underscore the importance of an integrative approach to managing ameloblastoma, considering histological subtypes, patient demographics, and long-term follow-up to ensure optimal outcomes.

In a retrospective study by Leite-Lima et al. 2023 [13], which analyzed 12 cases of UA treated over 20 years, conservative therapy was employed in all cases. This approach involved enucleation associated with chemical cauterization of the surgical wound using Carnoy’s solution and peripheral ostectomy of the cavity, with the extraction of associated teeth. The follow-up period ranged from 12 to 240 months, with recurrence observed in only one patient. Most cases corresponded to the mural subtype, recognized after complete enucleation of UA, underscoring the challenges of histological subtyping based on incisional biopsy. Despite the predominantly favorable outcomes with conservative therapy, one case of recurrence occurred in a patient with a mural subtype UA, emphasizing the importance of long-term follow-up.

A multivariate Cox regression analysis identified several key predictors of recurrence in patients with unicystic ameloblastoma. Tumor volume, bone cortex/soft tissue invasion, and root resorption were significant predictors, irrespective of the position and site characteristics of the tumor. A staging classification system can be used to predict patient prognosis based on primary tumor characteristics before initial surgery. A Stage I tumor would have a volume of ≤34.5 cm^3^ without bone cortex or soft tissue invasion, and Stage II tumor volume would be >34.5 cm^3^ or tumor invasion into the bone cortex/soft tissue. According to this staging system, 86 patients were classified as Stage I in Yang et al.’s study demonstrated a recurrence rate of 2.3% (2 cases), while 46 patients were classified as Stage II, with a recurrence rate of 34.8% (16 cases). Through the multivariate Cox regression analysis, tumor stage remained the primary significant predictor of recurrence. Stage II patients displayed a higher recurrence rate regardless of surgical control due to the lack of predictability of complete excision of the tumor once it spreads into the soft tissues. In addition, invasion of the cortical bone also results in poorer outcomes, deeming Stage II patients at higher risk of recurrence. Ultimately, variables such as root resorption, volume, position, and site characteristics were also identified as significant predictors of recurrence in the multivariate Cox regression analysis, highlighting the biological characteristics of the tumor that contribute to its aggressive nature and likelihood of recurrence, despite aggressive management [17].

## 4. Conclusions

In conclusion, unicystic ameloblastoma, though a relatively rare odontogenic tumor, presents significant clinical challenges due to its aggressive behavior and high recurrence rates. Radiographically, it displays both cystic and tumoral features, like unilocularity and root resorption, respectively. Histological subtyping is pivotal in guiding treatment decisions for UA, with conservative approaches demonstrating effectiveness in managing most cases. Tumor volume, root resorption, and cortical bone involvement as detected in radiographs, and soft tissue infiltration and ameloblastic cell differentiation, as detected by histopathological analysis, are all critical predictors of recurrence. As a result, a multidisciplinary diagnostic approach is imperative to achieve comprehensive surgical management in order to improve patient outcomes, starting from the general dentist and involving specialties like Oral Surgery, Oral Radiology, and Oral Pathology. Meticulous and regular long-term radiographic follow-up is essential to monitor and manage potential recurrences, particularly in cases with aggressive histological features such as the mural subtype. On the limitations of the study, the lack of tracking of the treatment process and follow-up has to be included, especially since mural unicystic ameloblastoma has a significant chance of recurrence.

## Data Availability

The original contributions presented in this study are included in the article. Further inquiries can be directed to the corresponding author.
